# Surgical Treatment of Gall-Stones

**Published:** 1902-04-12

**Authors:** 


### SURGICAL TREATMENT OF GALL-STONES.

A recent meeting of the Royal Medical and Chirurgical Society was engaged in a discussion on the surgical treatment of obstruction in the common bile duct caused by concretions. Mr. Mayo Robson communicated a paper on this subject in which he showed that in one out of every five or six cases in which it was necessary to operate for cholelithiasis the common bile duct had to be attacked. In 10 cases he had been able to manipulate the concretions back into the gall bladder and then remove them by cholecystotomy, and in 30 cases he had crushed them in the common duct. The latter method, however, was only suitable for soft stones, and for cases in which it was plainly possible to get rid of the fragments produced. In other cases choledochotomy was he thought the only operation on which reliance should be placed. By the improved methods now employed it was possible to complete the operation in 30 to 40 minutes. He regarded hæmorrhage and shock as the most important causes of death in these operations. He showed, however, that in his hands with increasing experience the mortality attending these operations was decreasing, and he thought it reasonable to assume that with due precautions the mortality of the operation should be reduced to 5 per cent. or under. Sir Dyce Duckworth, commenting upon this paper, said that in his experience none of the methods which had been proposed for dissolving or promoting the expulsion of gall-stones by medical means had been in any degree successful. But although gall-stones could not be removed by medical means, their formation could be prevented. The results of surgery in gall-stone cases were, he said, eminently satisfactory, and he added that a useful guide for the time of operation was a rise of temperature indicating inflammation—a little dictum in regard to which surgeons would probably not agree with him—inflammation being a most undesirable complication.

